# Clinical relevance of genotype–phenotype correlations beyond vascular events in a cohort study of 1500 Marfan syndrome patients with *FBN1* pathogenic variants

**DOI:** 10.1038/s41436-021-01132-x

**Published:** 2021-03-17

**Authors:** Pauline Arnaud, Olivier Milleron, Nadine Hanna, Jacques Ropers, Nadia Ould Ouali, Amel Affoune, Maud Langeois, Ludivine Eliahou, Florence Arnoult, Philippe Renard, Marlène Michelon-Jouneaux, Marie Cotillon, Laurent Gouya, Catherine Boileau, Guillaume Jondeau

**Affiliations:** 1 Université de Paris, LVTS, INSERM U1148, Hôpital Bichat-Claude-Bernard, Paris, France; 2 Centre National de Reference pour le Syndrome de Marfan et les Syndromes Apparentés, VASCERN HTAD European Reference Centre, AP-HP, Hôpital Bichat-Claude-Bernard, Paris, France; 3 Département de Génétique, AP-HP, Hôpital Bichat-Claude-Bernard, Paris, France; 4 Service de Cardiologie, AP-HP, Hôpital Bichat-Claude-Bernard, Paris, France; 5 Unité de Recherche Clinique, AP-HP, Hôpital Pitié- Salpêtrière, Paris, France; 6 Service d’explorations fonctionnelles, AP-HP, Hôpital Bichat-Claude-Bernard, Paris, France

## Abstract

**Purpose:**

Marfan syndrome (MFS) is a connective tissue disorder in which several systems are affected with great phenotypic variability. Although known to be associated with pathogenic variants in the *FBN1* gene, few genotype–phenotype correlations have been found in proband studies only.

**Methods:**

In 1,575 consecutive MFS probands and relatives from the most comprehensive database worldwide, we established survival curves and sought genotype–phenotype correlations.

**Results:**

A risk chart could be established with clinical and genetic data. Premature termination codon variants were not only associated with a shorter life expectancy and a high lifelong risk of aortic event, but also with the highest risk of severe scoliosis and a lower risk for ectopia lentis (EL) surgery. In-frame variants could be subdivided according to their impact on the cysteine content of fibrillin-1 with a global higher severity for cysteine loss variants and the highest frequency of EL surgery for cysteine addition variants.

**Conclusion:**

This study shows that *FBN1* genotype–phenotype correlations exist for both aortic and extra-aortic features. It can be used for optimal risk stratification of patients with a great importance for genetic counseling and personalized medicine. This also provides additional data for the overall understanding of the role of fibrillin-1 in various organs.

## INTRODUCTION

Marfan syndrome (MFS [MIM 154700]) is remarkable for its phenotypic variability and its evolving definition.^1,2^ Limiting the definition of MFS patients to individuals with heterozygous pathogenic variants in the *FBN1* gene has not reduced phenotypic variability as much as anticipated.^1^ Pathogenic variants in the *FBN1* gene have been associated with phenotypes ranging from normal (incomplete penetrance is rare but possible) to severe MFS in childhood with cardiovascular, ophthalmologic, skeletal, cutaneous, and neurologic features, and shortened life expectancy. This clinical variability and unpredictability remain limiting factors for personalized follow-up and effective genetic counseling.^3^ To date, more than 3,000 different MFS patients carrying more than 1,800 different pathogenic variants in the *FBN1* gene have been described, covering the full spectrum of phenotypes and variant types.^4^

MFS clinical variability is attributed to genotype–phenotype correlations and to modifying environmental effects and modifier-gene factors.^5^ However, few genotype–phenotype correlations have been reported:^6^ pathogenic variants within the neonatal region (corresponding to former exons 24–32, now 25–33) have been associated with more severe phenotypes, including neonatal MFS,^7^ while premature termination codon (PTC) variants (held responsible for haploinsufficiency) have been associated with more severe cardiovascular phenotypes than in-frame pathogenic variants (themselves being associated with a dominant negative effect).^8–11^ Variants changing the cysteine content in fibrillin-1 have been associated with more ophthalmologic manifestations.^7,12^ Most of these correlations were evaluated in studies of probands only, who usually display more severe phenotypes, thereby limiting the phenotypic spectrum and the possible identification of genotype–phenotype correlations.

Genotype–phenotype correlations are useful for genetic counseling and choosing medical therapy or surgery at the optimal moment, after weighing the individual’s benefit/risk ratio.^13^ In this study, we took advantage of a large population seen in a reference center, evaluated for all reported MFS-associated features in a standardized manner, including systematic familial screening, thus limiting a referral bias.

## MATERIALS AND METHODS

### Patients and clinical data

Patients are referred to our center because of clinically suspected MFS. Reasons for referral in children are usually great height, ectopia lentis, pectus deformation, and hypermobility. Adults are usually referred after the incidental discovery of thoracic aortic aneurysm, sometimes associated with skeletal features. Both adults and children come to the center as a result of familial screening. Much effort is put into the familial screening to be as exhaustive as possible in the evaluation of parents of patients.

During a 1-day hospitalization, patients undergo cardiologic evaluation including echocardiography with systematic and standardized measurement of aortic diameters, according to recommendations;^14^ ophthalmologic evaluation, including split-lamp examination; physical, skeletal evaluation (including spine and pelvis X-rays); skin examination; and recording of their complete medical history. Diagnoses are made during a multidisciplinary discussion the following week during which further investigations are prescribed when required. Follow-up at the center is proposed every 2–3 years, and yearly with the referring physician. Familial testing includes screening for the pathogenic variant, when it is known, or a complete clinical work-up, if no pathogenic variant is known. All clinical, imaging, and molecular genetics data are entered prospectively into a dedicated database. Data obtained at the last visit were used for this study.

### Molecular analysis

Genomic DNA was isolated from peripheral blood leukocytes using previously reported standard procedures.^15^ The *FBN1* gene (NM_000138.5) was originally screened by bidirectional sequencing of all coding exons and closely flanking intronic sequences (Big Dye terminators kit, ABI 3100 Genetic Analyzer, Applied Biosystems, Warrington, Cheshire, UK). Since 2015, the *FBN1* gene has been screened by next-generation sequencing, using a customized capture array (NimbleGen SeqCap®, Roche, France) comprising *FBN1* and 25 other genes known to be associated with thoracic aortic aneurysms. The capture array’s target regions spanned 132 kb. When possible, familial segregation of variants was investigated. Only likely pathogenic or pathogenic variants (classes 4 and 5 according to American College of Medical Genetics and Genomics–Association for Molecular Pathology [ACMG-AMP] recommendations) were considered for this study.^16^

### Variants categorization

Pathogenic variants in the *FBN1* gene (classes 4 and 5) were categorized either as in-frame or PTC. For the sake of clarity, the in-frame group comprised missense variants and small insertions/deletions of a number of nucleotides that was an exact multiple of 3. The PTC category was first comprised of nonsense variants and insertions/deletions of a number of nucleotides not divisible by 3 without ambiguity. The category was subsequently enlarged to include other molecular alterations, notably splice site variants. These lead to altered intron splicing through the activation of a nearby cryptic splice site (resulting either in partial exon exclusion or intron retention) or use of an alternative consensus 3’ or 5’ splice site (resulting in exon skipping). Splice site variants very often introduce a PTC resulting in the inactivation (loss of function/haploinsufficiency) of the mutated allele.^12,17^ In the case of in-frame exon skipping, as well as for exon deletion(s) or duplication(s), an altered (shortened or lengthened) peptide is expected to be produced. It can than either be misfolded and eliminated within the cell or secreted as an altered monomer expected to affect proper microfibril assembly. This assumption was previously explored experimentally by Liu et al. for in-frame exon deletions.^18^ They observed that the mutant messenger RNA (mRNA) was stable but that there was less fibrillin-1 matrix deposition sometimes associated with less protein synthesis. Furthermore, splice site variants may often lead to more than one outcome, so could lead to both skipping of the exon and use of a cryptic site, which further complicates the categorization. Targeted functional studies for each variant are necessary to achieve perfect categorization. These studies were performed and previously reported in an important subset of the MFS patients of this study.^19^ Indeed, we were able to show that the splice variants resulted in a significantly reduced amount of total mRNA in agreement with a mechanism of haploinsufficiency.^19^ Since functional data were not available for all molecular events, we cannot be sure that all splice variants result in haploinsufficiency. To consider this significant proportion of variants (more than 10% of our total population), we first tested independently each molecular subgroup that could produce PTCs: patients with a deletion or duplication of one or more exons or a pathogenic variant affecting the consensus splice site. The choice was made to pool these groups of variants together for our overall analysis in the absence of differences found between these patients and those with the other classic pathogenic PTC variants, which was also the strategy used in several previous genotype–phenotype studies in MFS.^20,21^

### Statistical analyses

Descriptive statistics are reported as mean ± SDs, unless stated otherwise. Categorical variables are reported as number and percentage rounded to the nearest integer. Kaplan–Meier estimated probabilities of survival, aortic events (aortic surgery, aortic dissection) as a function of age were compared with log rank tests. Statistical calculations were computed with Jump 7.0.1 software and R 3.4.0.^22^ Only global survival, aortic dissection risk, and aortic dissection or surgery risk were compared using tests of hypothesis. Voluntarily, no other formal statistical comparison has been performed, due to the fundamentally descriptive nature of the study and the multiple tests that would have been required otherwise, rendering *p* values meaningless.

## RESULTS

### Population

Among the 1,575 included patients carrying a pathogenic variant in the *FBN1* gene, mean age was 34.1 ± 17.8 years, 49% were males, 52% were probands (from 815 families), 20% underwent preventive aortic surgery, 9% experienced aortic dissection, and 25% underwent ectopia lentis surgery (Table 1). The global survival was 90% at 60 years. Compared with relatives, probands had more severe cardiovascular manifestations (Figure S1) and more frequent features in other systems (data not reported).

**Table 1:** MFS characteristic frequencies according to the type of pathogenic *FBN1* variant.

Characteristic	Total	PTC	In-frame pathogenic variants			
			Total	(−Cys)	(+Cys)	(noCys)
Population *N* (%)	1,575 (100)	627 (40)	948 (60)	352 (37)	114 (12)	482 (51)
Age year, mean (SD)	34.1 (17.8)	32.3 (16.3)	35.4 (18.7)	33.0 (17.9)	36.2 (20.0)	36.9 (18.8)
Males, *N* (%)	771 (49)	312 (50)	459 (48)	165 (47)	56 (49)	238 (49)
Familial, *N* (%)	1089 (69)	406 (65)	683 (72)	210 (59)	83 (73)	390 (81)
Proband, *N* (%)	815 (52)	355 (57)	460 (48)	212 (60)	53 (46)	195 (40)
Cardiovascular						
Aortic root diameter, mm, mean (SD)	37.8 (6.7)	37.8 (6.7)	37.0 (6.7)	37.3 (6.7)	34.7 (6.9)	37.2 (6.5)
Aortic dissection, *N* (%)	139 (9)	72 (11)	67 (7)	27 (8)	6 (5)	34 (7)
Preventive aortic surgery, *N* (%)	316 (20)	154 (25)	162 (17)	83 (24)	5 (4)	74 (15)
Mitral valve surgery, *N* (%)	93 (6)	41 (7)	52 (5)	21 (6)	3 (3)	28 (6)
Ophthalmologic						
Ectopia lentis, *N* (%)	896 (57)	334 (53)	562 (59)	261 (74)	78 (68)	223 (46)
Ectopia lentis surgery, *N* (%)	400 (25)	83 (13)	317 (33)	151 (43)	55 (48)	111 (23)
Skeletal						
Facial dysmorphia,^a^ *N* (%)	672 (43)	306 (49)	366 (39)	169 (48)	27 (24)	170 (35)
Teeth, *N* (%)	519 (33)	227 (36)	292 (31)	129 (37)	29 (25)	134 (28)
Arched palate, *N* (%)	991 (63)	443 (71)	548 (58)	226 (64)	56 (49)	266 (55)
Pectus, *N* (%)	725 (46)	347 (55)	376 (40)	178 (50)	20 (18)	180 (37)
Arachnodactyly, *N* (%)	609 (39)	419 (67)	432 (46)	202 (57)	67 (59)	230 (48)
Elbow extension <170°, *N* (%)	137 (9)	45 (7)	92 (10)	43 (12)	14 (12)	35 (7)
Flat feet, *N* (%)	619 (39)	295 (47)	324 (34)	135 (38)	21 (18)	168 (35)
Hypermobility, *N* (%)	74 (5)	45 (7)	29 (3)	8 (2)	1 (1)	20 (4)
Scoliosis, *N* (%)	712 (45)	327 (52)	385 (41)	159 (45)	18 (16)	208 (43)
Spondylolisthesis, *N* (%)	95 (6)	44 (7)	51 (5)	21 (6)	3 (3)	27 (6)
Acetabular protrusion, *N* (%)	417 (26)	188 (30)	229 (24)	110 (31)	17 (15)	102 (21)
Other						
Skin striae, *N* (%)	1038 (66)	459 (73)	579 (61)	325 (69)	40 (35)	304 (63)
Recurrent hernia, *N* (%)	86 (5)	44 (7)	42 (4)	17 (5)	5 (4)	20 (4)
Pneumothorax, *N* (%)	108 (7)	65 (10)	43 (5)	17 (5)	3 (3)	23 (5)
Dural ectasia, *N* (%)	293 (19)	151 (24)	142 (15)	67 (26)	7 (6)	68 (14)

Percentage were rounded to the nearest whole number.

*(−Cys)* pathogenic variant associated with a cysteine loss, *(+Cys)* pathogenic variant with a cysteine addition, *(noCys)* pathogenic variant with no cysteine modification, *MFS* Marfan syndrome, *PTC* premature termination codon variants.

^a^Facial dysmorphia (3/5: dolichocephaly, enophthalmos, downslanting palpebral fissures, malar hypoplasia, retrognathia).

### PTC vs. in-frame pathogenic variants

The 1,575 MFS patients carried 643 different pathogenic variants in the *FBN1* gene (classes 4 and 5) categorized either as “in-frame” (*n* = 379 [59%]) or “PTC” [*n* = 264 (41%]), figures in keeping with those reported in the UMD-FBN1 reference database.^4^ No significant differences were found between patients with a deletion or duplication of one or more exons (*n* = 53/1,575) or a pathogenic variant affecting the consensus splice site (*n* = 146/1,575) and those with the other classic pathogenic PTC variants. Therefore, we pooled these groups of variants together in the PTC category for our overall analysis.

**Fig. 1:**
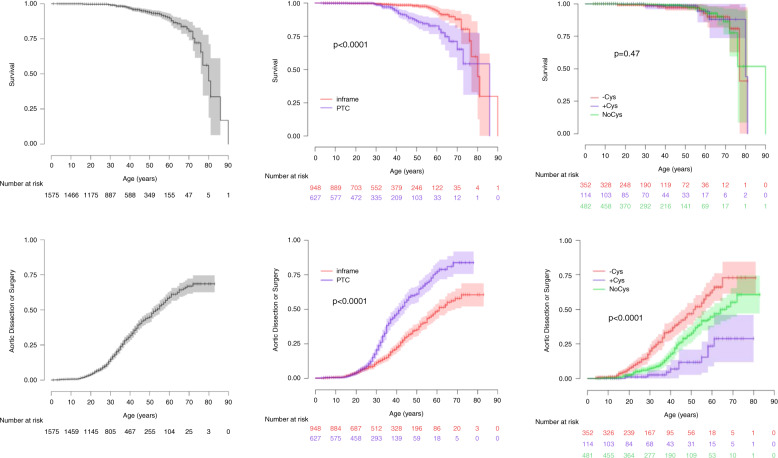
Kaplan–Meier estimated probabilities of the impact of the type of pathogenic variant in the *FBN1* gene as a function of age. Comparisons of survival (top), or aortic dissection or surgery (bottom). Left panel: all patients included in the study. Middle panel: patients with premature termination codon (PTC) (blue) vs. in-frame pathogenic variants (red). Right panel: patients with in-frame pathogenic variants associated with cysteine loss ([-Cys] in red), addition ([+Cys] in blue), or no fibrillin-1 protein cysteine content change ([noCys] in green).

Patients with PTC variants in the *FBN1* gene had more severe aortic phenotypes than those with in-frame variants (Table 1, Fig. 1), including a higher risk of aortic dissection (11% vs. 7%) or surgery (25% vs. 17%), and a larger mean aortic root diameter, despite younger age (suggesting faster aortic root dilation). In this group of patients, the life expectancy was shorter (*P* < 0.0001, Fig. 1) even when only probands were considered (*P* < 0.0001, data not shown). Mitral valve surgery was also more frequent (7% vs. 5%).

Similarly, all systemic manifestations were more frequent, with the notable exception of elbow extension limitation. Adults >20 years old were taller (183 vs. 180 cm; *P* < 0.001), despite similar weights (76.8 vs. 76.3 kg, *P* = 0.7), reflecting their typically longer long bones. In contrast, ectopia lentis and its surgery were less frequent in patients with pathogenic PTC variants (53% vs. 59%).

### Pathogenic in-frame variants

Cysteine is involved in disulfide-bridge formation. Therefore, variants affecting cysteine content (missense variants that substitute for a cysteine [+Cys] or that substitute a cysteine for another amino acid [−Cys]) are known to disrupt correct fibrillin-1 domain conformation and multimerization, most likely affecting its function and rendering the protein more vulnerable to proteolysis.^23,24^ The same consequences have also been observed for variants affecting residues within the highly conserved calcium-binding site sequences.^23,25^

In-frame (−Cys) variants, resulting in a disulfide-bond loss, were associated with particularly severe cardiovascular (more aortic dissection or surgery; *P* < 0.0001, Fig. 1), skeletal, and ophthalmologic phenotypes (Table 1). In contrast, in-frame (+Cys) variants were associated with fewer aortic events and with less severe forms of MFS, except for ophthalmologic features. Lastly, in-frame variants not modifying the cysteine content in fibrillin-1 (noCys variants) were associated with intermediate aortic risk and a skeletal phenotype broadly similar to (−Cys) variants but with a halved risk for ectopia lentis surgery. In this last group, no significant difference was found when considering variants affecting highly conserved amino acids involved in calcium-binding versus other in-frame variations (data not shown).

We also investigated whether the position of each cysteine in calcium-binding EGF-like domain (cysteines 1 to 6) and whether the different intradomain disulfide bonds (cysteine 1 and 3, cysteines 2 and 4, cysteines 5 and 6) were associated with different MFS severity levels. No clear differences could be evidenced (data not shown).

### Fibrillin-1 domains

Fibrillin-1 is a multidomain protein, comprising 47 epidermal growth factor-like domains, among which 43 are calcium-binding (cbEGF-like), 7 transforming growth factor-*β (*TGF-*β*)-binding protein domains (TB), 2 hybrid domains, and the N- and C-terminal domains (Table S1). We investigated whether in-frame pathogenic variants within these regions were associated with different MFS severity levels (Table S1). No clear differences could be evidenced.

### Localization of the pathogenic variants within the *FBN1* gene

#### Exons 24 to 32

**Fig. 2:**
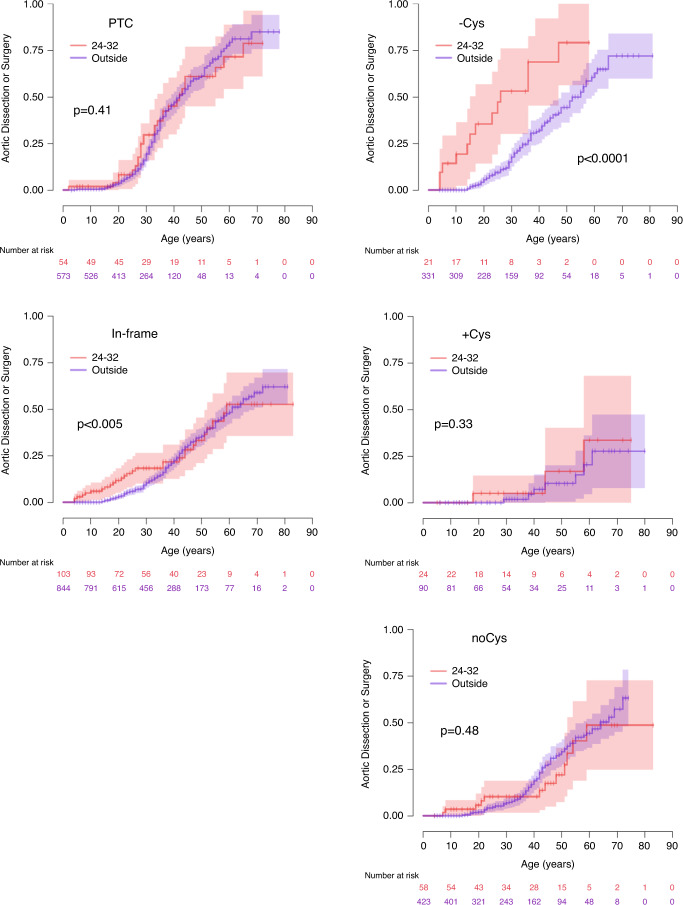
Kaplan–Meier estimated probabilities of the impact of a variant in the neonatal region (exons 24 to 32) vs. a variant located elsewhere in the *FBN1* gene on aortic risk (dissection or surgery) according to age. Comparison of variants located within the neonatal region (red) or elsewhere (blue) in the gene. Left panel: premature termination codon (PTC) (top), in-frame variants (bottom). Right panel: patients with in-frame pathogenic variants associated with (top) cysteine loss (-Cys), (middle) addition (+Cys), (bottom) no fibrillin-1 protein cysteine content change (noCys).

Overall, variants located between exons 24 and 32 are known to be associated with neonatal and severe adult forms of MFS through a dominant negative effect.^7^ However, in our study, PTC variants located in this region are not associated with particularly severe forms of the disease (Fig. 2). This reflects haploinsufficiency resulting from a systematic destruction of the incomplete mRNA produced by the cell’s nonsense-mediated decay (NMD) system.^19^ In contrast, in-frame pathogenic variants located between exons 24 and 32 had globally a more severe impact, especially concerning cardiovascular events, in particular for (−Cys) variants: in this subgroup, aortic and mitral valve surgery were more frequent and performed at a much earlier age, ectopia lentis surgery and skeletal features were also more frequent, including limited elbow extension (Table S2).

#### Exons 44 to 49

It has recently been reported that variants located within exons 44 to 49 are associated with more severe MFS.^26^ Indeed, the encoded sequence might regulate the bioavailability of endogenous TGF-β^27^ and explain disease severity when altered. We did not reproduce this finding and found no significant association when testing only variants located within these exons (data not shown).

### Sex effect

**Fig. 3:**
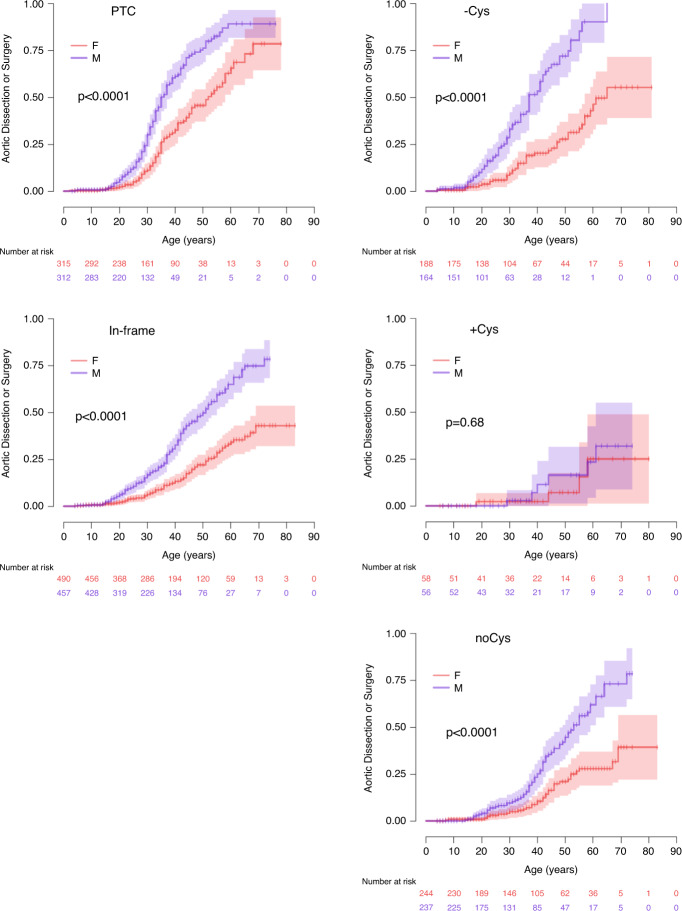
Kaplan–Meier estimated probabilities of a male (blue) vs. female (red) sex effect as a function of age in the different subgroups of pathogenic variants in the *FBN1* gene. Left panel: premature termination codon (PTC) (top), in-frame variants (bottom). Right panel: (top) patients with in-frame pathogenic variants associated with cysteine loss (-Cys), (middle) addition (+Cys), (bottom) no change in the cysteine content change (noCys).

**Fig. 4:**
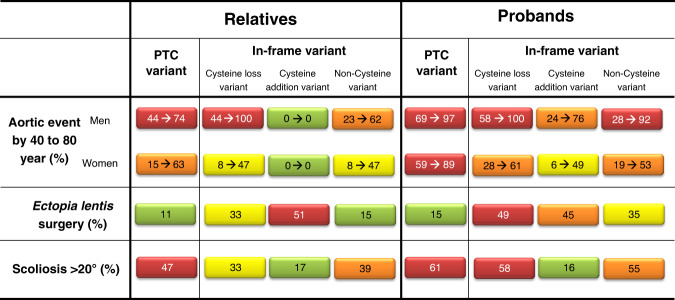
Risk chart for genetic counseling of patients with heterozygous *FBN1* pathogenic variants. Percentage of patients with aortic event by the age of 40 to 80 years, ectopia lentis, scoliosis >20° according to their variant groups. Risk levels are illustrated by different colors (red: very high risk, orange: high risk, yellow: intermediate risk, and green: low risk). PTC premature termination codon.

Males in our series had a more severe cardiovascular phenotype, as previously reported for the whole population,^28^ with more frequent aortic dissections and aortic surgeries in all subgroups (Fig. 3, Fig. 4), while the risk for ectopia lentis was similar. Finally and unexpectedly, skeletal features tended to be more frequent in women (scoliosis > 20°: 48% vs. 42%).

### Probands only

When only the probands were considered, similar trends were observed, including significantly lower survival (*P* < 0.0001), more aortic dissection (*P* < 0.001), and more aortic dissection or surgery (*P* < 0.0001) in patients carrying PTC variants when compared with in-frame variants. Differences in the clinical phenotype between probands carrying in-frame variants according to their effect on the cysteine content of the fibrillin-1 (−Cys; +Cys; noCys) were also significant for aortic dissection or surgery (*P* = 0.002). Of note, the genetic variants distribution slightly differs between probands and relatives, variants associated with a more severe phenotype being more frequent in probands: PTCs (44% vs. 36%) and –Cys (26% vs. 19%).

### Implications for genetic counseling and personalized patient follow-up

We propose a risk chart (Fig. 4) indicating disease severity according to the type of pathogenic variant. Severity is estimated with aortic event (aortic dissection or surgery) rates occurring by 40 and 80 years of age, lens surgery requirement and rate of severe scoliosis (>20°). Aortic event rate is ranging from 0% (relatives with in-frame [+Cys]) to 100% (men with in-frame [−Cys] pathogenic variants).

## DISCUSSION

We report survival, aortic event rate (dissection and surgery), and prevalence of extra-aortic features in a large population of MFS patients carrying a pathogenic variant in the *FBN1* gene and benefiting from modern care. We confirm that survival is improving compared with initial reports^29–33^ and we propose new tools to refine the prognosis in this population, i.e., strong genotype–phenotype correlations.

Pathogenic variants can be divided into PTCs and in-frame variants. PTCs are associated with haploinsufficiency, either through systematic destruction of PTC-containing transcripts by NMD system or altered processing and degradation of a truncated peptide. PTC variants in the *FBN1* gene were previously associated with more severe cardiovascular phenotypes than pathogenic in-frame variants.^10,20,26,34^ In keeping with these reports, PTCs in our study were associated with an 83% lifelong aortic event risk during life, and shorter life expectancy (Fig. 1). In contrast, ectopia lentis surgery was rarely required (13%) in the patients with a heterozygous PTC variant and half of these patients had severe scoliosis. No further subgroup delineation within this population was possible, as expected, because a unique molecular mechanism, haploinsufficiency, is involved.

The interpretation is more complex for patients with in-frame pathogenic variants. In these cases, the role of the pathogenic variant’s effect on the fibrillin-1 cysteine content and the importance of its location in the neonatal region in a subgroup of patients appear critical.

Fibrillins are the extracellular proteins with the highest content of cysteine residues (14%).^35^ These residues are important because they are implicated in disulfide bonds.^15^ These bonds are known to play a major role in fibrillin structure and function and represent the major sites of missense pathogenic variants found in Marfan syndrome patients.^4^ They are located within the tandem array of 4 epidermal growth factor-like (EGF-like), 43 cbEGF-like domains, the 7 TB domains, and the 2 hybrid domains. All these domains contain either 6 (EGF-like and cbEGF-like) or 8 to 9 (TB and hybrid) cysteine residues.

In-frame pathogenic variants leading to a cysteine loss at the protein level (−Cys) and thus resulting in loss of disulfide bridges within given fibrillin-1 modules were associated in our series with severe phenotypes: 73% lifelong risk of aortic dissection or surgery (Fig. 1), severe scoliosis frequency similar to that of PTCs (45%), and high risk for the need of ectopia lentis surgery (43%, Table 1). In-frame pathogenic variants leading to an additional cysteine (+Cys) were responsible for a mild cardiovascular phenotype (29% lifelong risk of an aortic event) and low severe scoliosis frequency (16%) but a similarly high risk for ectopia lentis surgery (48%). Lastly, in-frame pathogenic variants that do not change the cysteine content at the protein level (noCys) exhibited an intermediate cardiovascular phenotype (61% lifelong risk of surgery or dissection), severe scoliosis in 43%, with ectopia lentis surgery required for only a quarter of the patients.

When considering location of the variant within the gene and its consequence at protein level, we found no variant location in or out of a given protein module to be of importance. Only pathogenic variants within the “neonatal region” (exons 24 to 32) were associated with more severe phenotypes (Fig. 2). This is in keeping with previous reports.^7,36,37^ However, considering this subset of patients, we found that only the individuals from the (−Cys) group supported the most severe cardiovascular risk. Ophthalmologic and skeletal phenotypes were more severe in both (−Cys) and (+Cys) variants, whereas the (noCys) variants were only associated with a higher risk of ectopia lentis surgery. These observations suggest that altered conformation in the neonatal region has a major impact on the protein’s overall conformation. In line with this, when focusing on early onset events, 10 patients experienced an aortic event before the age of 10 years: 8 of 10 pathogenic variants were located in the neonatal region, while the remaining 2 were multiexon deletions (19–23 and 43–45), which might have been in-frame deletions.

The results of our study underscore the various cell-specific roles of fibrillin-1, which can be impacted differently by variants having different effects. Indeed, PTCs variants were associated with higher aortic risk, which means that haploinsufficiency leading to an expected smaller quantity of fibrillin-1 appeared critical in the aortic wall. Ectopia lentis being the most frequent feature in patients with (+Cys) or (−Cys) variants is in agreement with a structural role, in which case the dominant negative effect for (+Cys) or (−Cys) variants would be more deleterious than haploinsufficiency, because the protein in the extracellular matrix is a homopolymer.^38^ Lastly, skeletal features were less clearly related to a specific variant but patients with PTCs were taller.

These findings might be important for developing new therapeutics: a drug effectively containing one feature (e.g., decreasing aortic risk) might have limited impact on the severity of other features (e.g., ophthalmologic manifestations).

Beyond the variants in the *FBN1* gene, prognosis can be refined using the patient’s sex (Fig. 3). The greater aortic risk for males has long been known,^28,29^ but no clear explanation has been proposed. Herein, the sex impact was clear for all molecular subgroups, with earlier surgery reported for PTC-harboring patients, and the lifelong aortic risk for males being twice that of females with in-frame pathogenic variants.

Our approach was based on the study of a large population comprising both probands and their relatives carrying the familial *FBN1* pathogenic variant. Indeed, 48% of the population were relatives diagnosed during familial screening. Contrary to association studies in which relatedness needs to be considered if relatives are included in the study population, the investigation of genotype–phenotype correlations only warrants the presence in the study population of a pathogenic variant within given a gene. Therefore, both probands and their relatives can be pooled for these types of studies. This is important in the study of rare diseases for which large sample sizes are difficult to obtain and thus statistical significance is hard to reach. Another reason to include relatives in genotype–phenotype correlation studies is the accepted fact that probands represent the most severe end of the phenotype spectrum, thus introducing a severity bias (as observed in this study).

These results also have implications for patient care. Preventive therapy with β-blockers and restricted exercise should be proposed regardless of the aortic diameter in the high aortic risk groups (PTCs or [−Cys] variants).^14,39^ This may be proposed only in patients with dilated aorta carrying in-frame (noCys) variants, particularly females, and those carrying (+Cys) variants. Because preventive surgery is very likely to be necessary at some time during the lives of patients with PTC variants, decision to intervene should be facilitated for them. In contrast, avoiding surgery should be the aim in patients with low aortic risk (i.e., +Cys variants) in whom medical therapy would produce maximal benefit.^40^ Future studies to evaluate medical treatment effect on aorta diameter evolution should focus on patient with PTC and (–C) variants. It should be noted that differences in the rate of aortic dissection reported here compared with that reported previously by Milleron et al.^41^ reflect the different populations studied. Both are dealing with patients with *FBN1* pathogenic variants seen in the reference center; however, in the present study this was the only selection criterion, whereas only patients without surgery or dissection at the time of the first visit and seen at least twice in the reference center were included in the previous paper. The difference in aortic event rate between the two populations therefore underscores the benefit of early diagnosis and up-to-bdate care, including exercise limitation, β-blocker therapy, regular follow-up, and timely prophylactic aortic root surgery. Our results also have major consequences on genetic counseling of patients with a pathogenic *FBN1* variant. First, relatives are in general less severely affected in all organ systems compared to the probands. Second, a recurrent question concerns the impact on offspring, which can sometimes lead to consideration of prenatal diagnosis. Our findings may help more clearly answer the questions of future parents relative to the severity of the disease in their offspring, although it should be kept in mind that these numbers are merely statistics and thus not a guarantee, because variability is observed within each group.

This study has several limitations. The single-center recruitment means a referral bias remains possible, despite systematic familial screening and national recruitment. Because the MFS referral center receives mainly outpatients, very severe neonatal MFS forms are underrepresented, and parents who died before diagnosis were not included. The prevalence of various features is provided according to age only for cardiovascular features, which are the most dependent on age. Ectopia lentis is usually present very early in life, but the skeletal features appear and evolve during childhood. As 15% of the population studied is below 15 years of age, this may have led to a slight underestimation of the prevalence of skeletal features. Although the number of patients studied is large for a rare disease, new genotype–phenotype correlations might be found in even larger populations.

Herein, we reported new MFS genotype–phenotype correlations, which are of great importance for genetic counseling, personalized medicine, and our overall understanding of the role of fibrillin-1 in various organs.

## Web Resources

OMIM, https://www.omim.org/.

## Supplementary information

Supplementary Information

## Data Availability

The data are available upon request.
