# Supplementary material for: Clinical relevance of genotype–phenotype correlations beyond vascular events in a cohort study of 1500 Marfan syndrome patients with FBN1 pathogenic variants
Source: Genet Med. 2021 Mar 17;23(7):1296–304. doi: 10.1038/s41436-021-01132-x (PMC8257477; doi:10.1038/s41436-021-01132-x)

Supplemental Appendix

Table S1. Phenotype Frequencies According to the Protein Domain(s) Affected by In-frame Pathogenic Variants.

Characteristic	Domain Affected			
	cbEGF-like	Hybrid	TB	EGF-like
N (**%**)	663 (**70**)	99 (**10**)	98 (**10**)	47 (**6**)
Age yr, mean (SD)	34.7 (18.0)	34.3 (18.3)	37.1 (20.1)	32.7 (18.0)
Males, N (%)	315 (48)	50 (50)	48 (49)	23 (49)
Familial, N (%)	463 (70)	83 (83)	72 (73)	32 (68)
Proband, N (%)	338 (51)	33 (33)	48 (49)	24 (51)
Cardiovascular				
Aortic root diameter, mean (SD)	37.6 (6.6)	36.2 (6.7)	37.1 (6.6)	35.7 (7.3)
Aortic dissection, N (%)	48 (7)	9 (9)	3 (3)	3 (6)
Preventive aortic surgery, N (%)	122 (18)	15 (15)	15 (15)	4 (9)
Mitral valve surgery, N (%)	42 (6)	6 (6)	2 (2)	1 (2)
Ophthalmologic				
Ectopia lentis, N (%)	390 (59)	69 (70)	46 (47)	31 (66)
Lens ablation, N (%)	218 (33)	43 (43)	24 (24)	24 (30)
Skeletal				
Facial dysmorphia, N (%)	260 (39)	38 (38)	34 (35)	12 (26)
Teeth, N (%)	212 (32)	33 (33)	23 (23)	14 (30)
Arched palate, N (%)	380 (57)	61 (61)	53 (54)	27 (57)
Pectus, N (%)	248 (37)	37 (37)	37 (38)	17 (36)
Arachnodactyly, N (%)	361 (54)	44 (44)	41 (42)	15 (32)
Elbow extension <170°, N (%)	59 (9)	7 (7)	12 (12)	10 (21)
Flat feet, N (%)	229 (35)	35 (35)	35 (36)	11 (23)
Hypermobility, N (%)	24 (4)	2 (2)	2 (2)	0 (0)
Scoliosis, N (%)	287 (43)	29 (29)	36 (37)	12 (19)
Spondylolisthesis, N (%)	40 (6)	4 (4)	7 (7)	0 (0)
Acetabular protrusion, N (%)	172 (26)	13 (13)	26 (27)	9 (19)
Other				
Skin striae, N (%)	421 (63)	57 (57)	53 (54)	19 (40)
Recurrent hernia, N (%)	30 (5)	5 (5)	3 (3)	2 (4)
Pneumothorax, N (%)	29 (4)	6 (6)	4 (4)	2 (4)
Dural ectasia, N (%)	106 (16)	14 (14)	11 (11)	3 (6)

EGF-like, epidermal growth factor-like domains; cbEGF-like, calcium-binding EGF like domains; TB, transforming growth factor-*β*–binding protein-like domains.

Table S2. MFS phenotypes associated with in-frame pathogenic variants according to their effect on cysteine content and localization within the neonatal hot spot region or the rest of the gene.

Characteristic	(-Cys) *		(+Cys) *		(noCys) *	
	24-32†	Other	24-32†	Other	24-32†	Other
Population N (**%**)	21 (**1**)	332 (**21**)	24 (**2**)	90 (**6**)	59 (**4**)	423 (**27**)
Age yr, mean (SD)	31.1 (18.1)	33.1 (14.9)	37.3 (20.3)	35.9 (20.0)	36.1 (19.4)	37.1 (18.7)
Males, N (%)	10 (48)	155 (47)	10 (42)	46 (51)	24 (41)	214 (51)
Familial, N (%)	3 (14)	207 (62)	18 (75)	65 (72)	41 (69)	349 (83)
Proband, N (%)	20 (95)	192 (58)	13 (54)	40 (44)	35 (59)	160 (38)
Cardiovascular						
Aortic root diameter mm, mean (SD)	40.0 (3.1)	37.7 (6.9)	36.0 (1.0)	34.3 (0.8)	37.3 (5.4)	37.2 (6.6)
Aortic dissection, N (%)	4 (19)	23 (7)	2 (8)	4 (4)	2 (3)	32 (8)
Preventive aortic surgery, N (%)	11 (52)	72 (22)	1 (4)	4 (4)	11 (19)	63 (15)
Mitral valve surgery, N (%)	8 (38)	13 (3.9)	3 (13)	0 (0)	6 (10)	22 (5)
Ophthalmologic						
Ectopia lentis, N (%)	16 (76)	245 (74)	12 (50)	66 (73)	33 (56)	190 (45)
Lens ablation, N (%)	14 (67)	137 (41)	8 (33)	47 (52)	26 (44)	85 (20)
Skeletal						
Facial dysmorphia†, N (%)	15 (71)	154 (47)	4 (17)	23 (26)	26 (44)	144 (34)
Teeth, N (%)	10 (48)	119 (36)	8 (33)	21 (23)	19 (32)	115 (27)
Arched palate, N (%)	18 (86)	208 (63)	12 (50)	44 (49)	34 (58)	232 (55)
Pectus, N (%)	13 (62)	165 (48)	8 (33)	12 (13)	16 (27)	164 (39)
Arachnodactyly, N (%)	18 (86)	184 (56)	8 (33)	35 (39)	28 (47)	202 (48)
Elbow extension <170°, N (%)	9 (43)	34 (10)	2 (8)	12 (13)	4 (7)	31 (7)
Flat feet, N (%)	9 (43)	126 (38)	4 (17)	17 (19)	22 (37)	146 (35)
Hypermobility, N (%)	0 (0)	8 (2)	1 (4)	0 (0)	2 (3)	18 (4)
Scoliosis, N (%)	16 (76)	143 (43)	4 (17)	14 (16)	25 (42)	183 (43)
Spondylolisthesis, N (%)	0 (0)	21 (6)	2 (8)	1 (1)	0 (0)	27 (6)
Acetabular protrusion, N (%)	12 (57)	98 (30)	5 (21)	12 (13)	12 (20)	90 (21)
Other						
Skin striae, N (%)	13 (62)	222 (67)	7 (29)	33 (37)	36 (61)	268 (63)
Recurrent hernia, N (%)	3 (14)	14 (4)	0 (0)	5 (6)	3 (5)	17 (4)
Pneumothorax, N (%)	3 (14)	14 (4)	0 (0)	3 (3)	2 (3)	21 (5)
Dural ectasia, N (%)	10 (48)	57 (17)	3 (13)	4 (4)	11 (19)	57 (13)

* (-Cys): pathogenic variant associated with a cysteine loss; (+Cys): pathogenic variants with a cysteine addition; (noCys): pathogenic variant with no cysteine modification; 24-32†: neonatal region (exons 24 to 32).

Figure S1. Kaplan–Meier Estimated Probabilities of Events Occurring in Probands (red) vs. Non-Probands (blue), According to Age.

Comparisons of survival (top), aortic dissection (middle), and aortic dissection or aortic surgery (bottom).

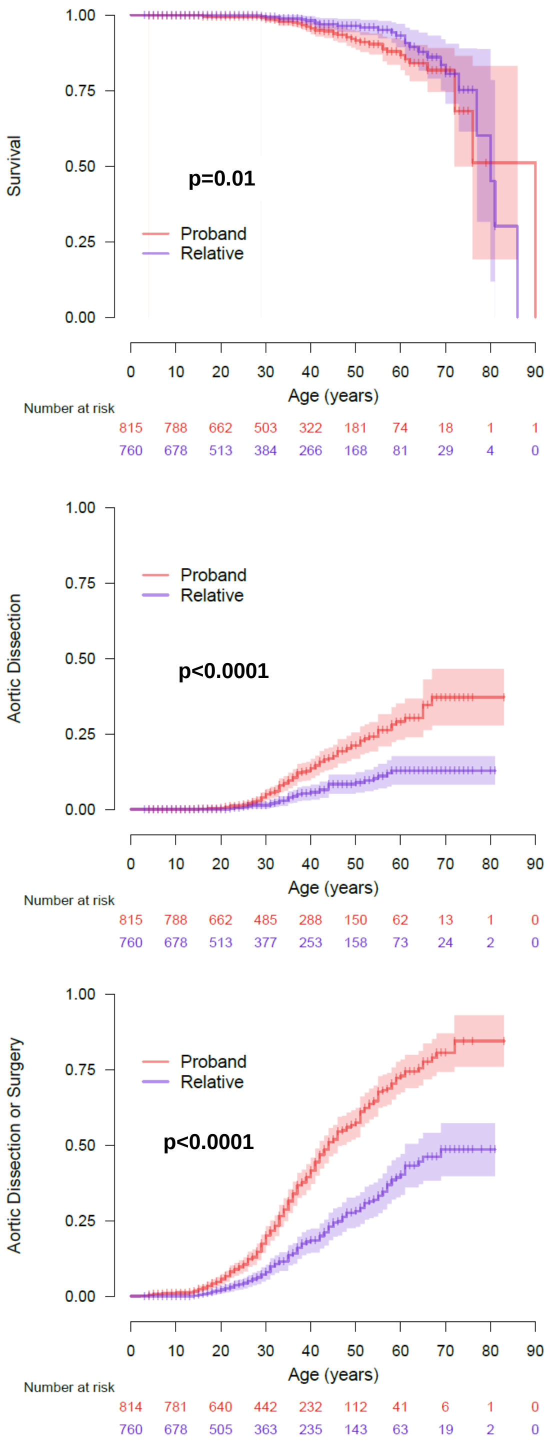

Supplement: Supplementary file 1 — Supplementary Information [file 41436_2021_1132_MOESM1_ESM.pdf]
